# Orally Administrable Therapeutic Nanoparticles for the Treatment of Colorectal Cancer

**DOI:** 10.3389/fbioe.2021.670124

**Published:** 2021-07-07

**Authors:** Kangkang Ying, Bingjun Bai, Xing Gao, Yuzi Xu, Hangxiang Wang, Binbin Xie

**Affiliations:** ^1^The First Affiliated Hospital, Zhejiang University School of Medicine, Hangzhou, China; ^2^National Health Commission (NHC), Key Laboratory of Combined Multi-Organ Transplantation, Hangzhou, China; ^3^Key Laboratory of Organ Transplantation, Research Center for Diagnosis and Treatment of Hepatobiliary Diseases, Hangzhou, China; ^4^Department of Medical Oncology, Sir Run Run Shaw Hospital, School of Medicine, Zhejiang University, Hangzhou, China; ^5^Department of Colorectal Surgery, Sir Run Run Shaw Hospital, School of Medicine, Zhejiang University, Hangzhou, China; ^6^Department of Oncology, The Second Affiliated Hospital of Soochow University, Suzhou, China; ^7^Department of Oral Implantology and Prosthodontics, The Affiliated Hospital of Stomatology, School of Stomatology, Zhejiang University School of Medicine, Hangzhou, China; ^8^Key Laboratory of Oral Biomedical Research of Zhejiang Province, Hangzhou, China

**Keywords:** oral administration, drug delivery, nanoparticles, colorectal cancer, cancer therapy

## Abstract

Colorectal cancer (CRC) is one of the most common and lethal human malignancies worldwide; however, the therapeutic outcomes in the clinic still are unsatisfactory due to the lack of effective and safe therapeutic regimens. Orally administrable and CRC-targetable drug delivery is an attractive approach for CRC therapy as it improves the efficacy by local drug delivery and reduces systemic toxicity. Currently, chemotherapy remains the mainstay modality for CRC therapy; however, most of chemo drugs have low water solubility and are unstable in the gastrointestinal tract (GIT), poor intestinal permeability, and are susceptible to P-glycoprotein (P-gp) efflux, resulting in limited therapeutic outcomes. Orally administrable nanoformulations hold the great potential for improving the bioavailability of poorly permeable and poorly soluble therapeutics, but there are still limitations associated with these regimes. This review focuses on the barriers for oral drug delivery and various oral therapeutic nanoparticles for the management of CRC.

## Introduction

Colorectal cancer (CRC) is the third leading cause of cancer incidence and the second leading cause of cancer-related mortality, with 1.80 million new cases and approximately 900,000 deaths annually (Bray et al., 2018). Approximately 74% of CRC cases occur in the colon whereas 26% of CRC cases are reported in the rectum (Liu Y. et al., 2018). CRC is a malignant tumor derived from glandular cells and epithelial cells of the colon or rectum. Treatment approaches for CRC include surgical resection, local ablation, neoadjuvant chemotherapy, intra-arterial chemotherapy, adjuvant chemotherapy, radiotherapy, targeted therapy and immunotherapy (Punt et al., 2017; Dekker et al., 2019; Franke et al., 2019).

Chemotherapy remains a mainstay regimen for the management of CRC. In particular, the use of nanoparticles (NPs) has been widely successful for pharmaceutical delivery of active compounds (Pavitra et al., 2019). This approach enables higher intratumoral drug delivery and reduced systemic toxicity than free drug dosages (Omar et al., 2007; Pinto, 2010). Previous studies have explored various nanoparticle-based systems, such as liposomes, micelles, polymeric NPs, phytosomes, dendrimers, gold NPs, and magnetic NPs with the aim of improving drug aqueous solubility and achieving disease-specific drug delivery (Cisterna et al., 2016; Wong K. E. et al., 2019).

The oral route of drug administration is one of the most preferred approaches because of several advantages including: (1) It is the simplest, most convenient and safest way of drug administration; (2) It is painless and can be self-administered, thus improving patient compliance; (3) It is convenient for patients to repeat the medication for a long time; (4) No special knowledge or special supplies are required; (5) No sterile precautions are required; (6) Extra economic cost of adjuvant drugs is low; and (7) An acute reaction of the drug is less likely to happen (Wang S. et al., 2015; Griffin et al., 2016; Jain and Jain, 2016; Alavian and Shams, 2020). Furthermore, a wide range of drug molecules, from small molecular compounds to large biomacromolecules, are tolerable in this administration route. More importantly, oral route is highly attractive for local drug delivery, especially for many diseases localized in the gastrointestinal tract (GIT).

Despite significant advances made in this field, oral drug administration for CRC therapy remains a big challenge. The efficacy of oral formulations has been generally compromised by several limitations, such as drug poor solubility, poor permeability, rapid degradation in the GIT, and the inability to penetrate and low adsorption across the mucosal barrier (Hua, 2014). In this review, we provide an updated summary and prospects of oral drug delivery systems for treatment of CRC.

## Background of Colorectal Cancer

Colorectal cancer (CRC) is a common and one of the leading causes of cancer-related deaths. High incidence of CRC is associated with lifestyle changes (Brody, 2015). Advances in colorectal cancer screening approaches significantly reduce the incidence and mortality of CRC in developed countries. In addition, the mortality and incidence of CRC can be significantly reduced by implementing population-based screening programs (Feng et al., 2019). However, in some developing countries, incidence and death rates of CRC continue to rise due to westernized lifestyles, intake of low-fiber and high fat diet, smoking, heavy alcohol intake, obesity and reduced physical activity among the population (Wong M. C. et al., 2019; Siegel et al., 2020). CRC is characterized by late diagnosis and poor treatment due to high rates of metastatic burden as well as chemoresistance (Arvelo et al., 2015).

**FIGURE 1:**
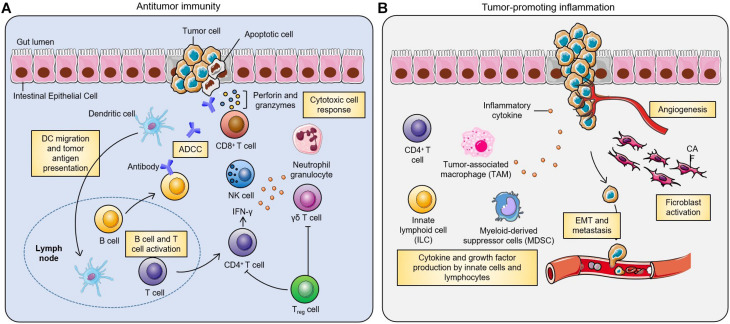
Inflammation is a double-edged sword in development of colorectal cancer (West et al., 2015). Inflammation plays a critical role in the pathogenesis of CRC. **(A)** In anti-tumor immunity, dendritic cells can recognize and present tumor antigens and activate tumor-specific B cells and T cells. Traditionally, effective anti-tumor immunity is mainly mediated by CD4^+^ T cells, CD8^+^ T cells and natural killer cells (NK cells). There is growing evidence that γδ T cells are also involved in anti-tumor immunity, and that Treg cells can restrain this process. **(B)** In addition, inflammation can also promote tumor development. CD4^+^ T cells, innate lymphoid cells and tumor-associated macrophages in the tumor microenvironment can induce the production of a variety of inflammatory cytokines, which lead to tumor proliferation, invasion, angiogenesis, epithelial-mesenchymal transition and metastasis.

Colon and rectum play important roles in digestion by aiding absorption of water, minerals, nutrients and storage of wastes. CRC develops in a stepwise manner. Mutations of critical genes in neoplastic cells acquire survival advantage over normal epithelial cells (Vermeulen et al., 2013). These mutations induce the precancerous tissue to evolve to adenomas and then to carcinomas. Molecular mechanisms involving the occurrence and development of CRC are summarized previously (Markowitz and Bertagnolli, 2009). These mutations happen sequentially during progression of CRC and are associated with the stages of tumor development and histopathology (Fearon and Vogelstein, 1990). Notably, chronic intestinal inflammation is a major risk factor. Figure 1 summarizes the relationship between inflammation and CRC (West et al., 2015). Inflammation has many opposing effects on the development of CRC. Inflammation can induce anti-tumor effect in intestines, which is mediated by several immune cells including dendritic cells that recognize and present antigens, B cells and T cells. In addition, non-specific immunity orchestrated by natural killer cells, neutrophils, Treg cells and γδ T cells contributes to anti-tumor effects of inflammation. On the other hand, inflammation can promote tumor development. Tumor-associated immune cells in the tumor microenvironment (TME) trigger the production of several inflammatory factors which promote the proliferation, invasion, epithelial-mesenchymal transition, metastasis, angiogenesis and other processes in tumor cells hence enhancing the progression of tumors. In addition to environmental and genetic factors, studies showed that gut microbiota play a key role in shaping the inflammatory environment and promoting development of CRC (Brennan and Garrett, 2016).

Traditional chemotherapy for treatment of CRC has limited efficacy due to its toxicity and side effects on normal tissues (Prados et al., 2013). Therefore, CRC treatments are in urgent need of safe and effective new therapies. NP-mediated oral systems can enhance the drug efficacy and reduce side effects over their free drug forms (Hu et al., 2010). Use of NPs as delivery vehicles holds several advantages: (1) NP encapsulation can improve drug solubility and stability under harsh GIT conditions; (2) NP formulation could extend the half-life of drug payloads in the blood circulation (Fearon and Vogelstein, 1990); (3) NPs have enhanced permeability and retention (EPR) effect in tumor lesions, hence passive targeting capacity compared with free drugs, which is expected to augment intratumoral drug accumulation which is essential for high efficacy (Wang et al., 2012); (4) some NP strategies have been developed to subvert drug resistance mechanisms in cancer cells, thus significantly reducing the concentration of drugs required for treatment (Beretta and Cavalieri, 2016); (5) some therapeutics such as nucleic acids can be formulated in NPs to improve the stability and avoid *in vivo* degradation (Mokhtarzadeh et al., 2019); (6) NPs can be designed to target specific sites and encapsulate multiple drugs to produce good therapeutic effects (Banerjee and Sengupta, 2011; Blanco et al., 2011); and (7) toxicity can be reduced by reducing systemic drug exposure. Currently, numerous NPs that target cancer cells or TME have been examined for efficacy testing against preclinical CRC models (Table 1); however, most of them are not clinically approved.

**TABLE 1:** Summary of nanotechnology-based systems for CRC.

Formulation/NPs platforms	Drugs	Target/indication	References
HACTNp	5-FU	Cancer cells	Jain and Jain, 2008
SLNPs containing 5-FU	5-FU	Cancer cells	Kamel et al., 2017; Entezar-Almahdi et al., 2020
5-FU/PEG-PBLG	5-FU	Cancer cells	Li et al., 2008
5-FU-loaded PCL NPs	5-FU	Cancer cells	Liu et al., 2012
5-FU/GSH-GNPs	5-FU	Cancer cells	Zhao et al., 2018
pHLNps-5-FU	5-FU	Cancer cells	Udofot et al., 2015
Xyl-SA/5-FUSA	5-FU	Cancer cells	Sauraj, Kumar et al., 2019
EGF-HMSNs-5-FU	5-FU	Cancer cells	Chen et al., 2015
5-FU ZGGO@SiO2@LRM	5-FU	Cancer cells	Wang Z. H. et al., 2019
Chitosan NPs	5-FU, leucovorin	Cancer cells	Li P. et al., 2011; Smith et al., 2018
Nano-Folox	5-FU, Oxaliplatin	Cancer cells	Guo et al., 2020
Chitosan-HA-Oxa NPs	Oxaliplatin	Cancer cells	Jain et al., 2010
Oxaliplatin encapsulated in chitosan-coated alginate microspheres	Oxaliplatin	Cancer cells	Urbanska et al., 2012
PEG-liposomal L-oHP	Oxaliplatin	Cancer cells	Yang et al., 2011
Liposome-Embedding Silicon Microparticle	Oxaliplatin	Cancer cells	Cevenini et al., 2020
Nanoscale coordination polymer (NCP) core-shell particles	Oxaliplatin, DHA	TME	Duan et al., 2019
CPX-1	Irinotecan HCl	Cancer cells	Batist et al., 2009
pH-Responsive PEG-Shedding and Targeting Peptide-Modified Nanoparticles	Irinotecan, miR-200	Cancer cells	Juang et al., 2019
Lipid bilayer-coated MSNP carrier	Irinotecan	Cancer cells	Liu et al., 2019
Liposomal irinotecan (Lipo-IRI)	Irinotecan	Cancer cells	Huang et al., 2019
LE-SN38	SN38	Cancer cells	Lei et al., 2004
SN38 (LA-SN38)-loaded NPs	SN38	Cancer cells	Cheng et al., 2019
CD133Ab-NPs-SN-38	SN38	Cancer cells	Ning et al., 2016
nSN38, nCUR	SN38, curcumin	Cancer cells	Han et al., 2019
PLGA-PTX	Paclitaxel	Cancer cells	Zhong et al., 2019
Paclitaxel-loaded magnetic nanocarriers	Paclitaxel	Cancer cells	Dehvari et al., 2016
Celecoxib-containing Hap-Cht NPs	Celecoxib	Cancer cells	Venkatesan et al., 2011
QT-SLNPs	Quercetin (QT)	Cancer cells	Li et al., 2009
PLGA NPs loaded with DCPIP	DCPIP	Cancer cells	de Haan et al., 2006; Cabello et al., 2009
Chitosan NPs	Gemcitabine, curcumin	Cancer cells	Hosseinzadeh et al., 2012; Chuah et al., 2013
WGA-conjugated PLGA NPs loaded with Pac	Pac	Cancer cells	Huh et al., 2009; Wang et al., 2010; Hosseinzadeh et al., 2012; Chuah et al., 2013
Aspirin-loaded nanoexosomes	Aspirin	Cancer cells	Tran et al., 2019
A33Ab-US-Exo/Dox	Doxorubicin	Cancer cells	Li et al., 2018
EGFR-targeted evodiamine NPs	Evodiamine	Cancer cells	Li et al., 2019
miR-139-5p-EpCAM Apt-HSPC/DOTAP/Chol/DSPE-PEG2000-COOH nanoparticles, MANPs	miR-139-5p	Cancer cells	Zhao et al., 2019
Chol-butyrate SLNP formulation	Butyric acid	Cancer cells, Endothelial cells	Brioschi et al., 2008; Minelli et al., 2012
PEG-PLGA-endostar	Endostar	Tumor vessels	Chen and Hu, 2011
Hafnium oxide nanoparticles (NBTXR3)		Cancer cells	Marill et al., 2019
Silver nanoparticles (AgNPs)		Cancer cells	Gurunathan et al., 2018
PEG-AuIONs		MRI	Kumagai et al., 2010
Silica-coated NPs coated with an iron oxide (IO) core and containing an organic dye (MFSNs)		MRI	Yoon et al., 2005

## Barriers for Oral Administration of Anticancer Nanotherapeutics

Drugs previously used for CRC treatment are either invasive or non-selective. Some nanosystems that allow oral administration have been developed. Oral administration is able to increase patient compliance and decreases side effects. An ideal orally administered drug for CRC therapy should reach the colon and rectum, specifically accumulate at the lesion sites, and also be deliverable to metastatic lesions through systemic circulation (Hua, 2014). However, NPs take long time before approaching the colon and rectum, including gastropore, which limits oral efficacy (Wahlgren et al., 2019).

**FIGURE 2:**
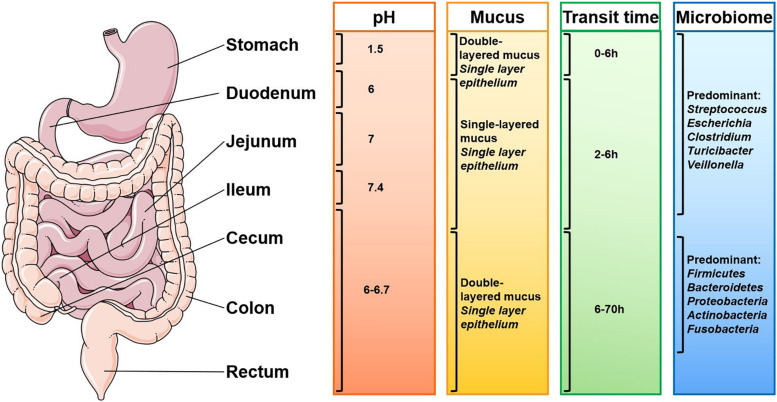
Gastrointestinal physiological factors that influence oral drug delivery (adapted from Hua, 2020). The complex gastrointestinal (GI) environment of human beings and the differences in different individuals bring challenges to the delivery of nanoparticles. The pH of the GI tract, composition of the mucosa, microorganisms and enzymes, and the residence time of the drugs in different parts of the GI tract all affect the drug delivery effect.

Oral drug delivery poses three barriers occurred in the GIT (O’Driscoll et al., 2019): enzymatic barrier that may cause degradation of drug molecules, mucus barrier that may trap hydrophilic molecules in the mucus, and membrane barrier that may prevent drugs from entering enterocytes. In this section, we discuss the limitations regarding the NP delivery. The transit time of NPs along the GIT is influenced by several physiological factors including pH, mucosal layer, and microbiota (Figure 2; Hua, 2020). These factors also affect drug adsorption.

### Stability

Upon orally administered, the stability of NPs and relevant drug payloads remains a crucial factor because of the acidic pH conditions in the stomach (1.0–2.5). Ideal oral NPs should be stable in the gastric fluid for effective delivery to the small intestine, colon and rectum (Homayun et al., 2019). After leaving the stomach, NPs are transported along the duodenum, jejunum, and ileum (pH value changes from 6 to 7.4), then through the caecum (pH = 5.7) and rectum (pH = 6.7) (Fallingborg, 1999). In addition, gastrointestinal protease and lipase act on drugs, thus affecting their activity. Liposomes are susceptible to combined effects of gastric acid, bile salt and pancreatic lipase in the GIT, resulting in reduced concentration of intact liposomes and payload leakage (Hu et al., 2013). Moreover, the gut especially the colon is rich in microbiome which may lower drug activity. Furthermore, hydrolases and metabolic enzymes in bacteria may degrade drugs and affect their stability (Rowland, 1988; Sinha and Kumria, 2001).

### Permeability

NPs are absorbed from the intestinal lumen into the bloodstream of target tissues through endocytosis/pinocytosis or via carrier-mediated transport. Thus, the gastrointestinal mucosal barrier is an additional obstacle that impairs the effective uptake of NPs. Small intestine is the most permeable region of GIT because of its larger absorptive surface and leaky paracellular channels (Ritschel, 1991). Therefore, oral NPs need to be targeted to the small intestine for maximum absorption. In addition, drugs encounter hepatic barrier after entering blood vessels of intestinal epithelium (Homayun et al., 2019).

### Transport

NPs ensures specific drug delivery to the lesion site of the colon and rectum. There are three major pathways for NPs to move from the apical side to the basal side of cells, including: paracellular pathway through tight junctions, transenterocytic pathway and M-cell-mediated pathway (Yu et al., 2016). Transenterocytic pathway comprises uptake, endocytosis, transport within the cell and exocytosis from the interior of the cell, thus it is the most effective pathway (Reinholz et al., 2018). However, in the endolysosomal uptake pathway, NPs are trapped successively in endosomes and lysosomes, and they are degraded by enzymes or infinitely accumulated in the lysosomes, thus affecting their transport.

To overcome these barriers, traditional NPs need be rationally re-engineered. For example, coating the particle surface with a suitable layer or storing them in an airtight container may help to augment the stability of therapeutic NPs against degradation by the harsh conditions in GIT (Xu L. et al., 2018). Moreover, surface modification of nanoparticles not only increases their stability, adhesion and permeability, but also improves their sustained and controllable drug release properties.

Another aspect through which the transport of NPs is impaired is via the degradation by lysosomes in epithelial cells. Some strategies have been developed to prevent endo/lysosomal damage (Martens et al., 2014; Selby et al., 2017), such as surface modification of NPs with endosomal escape agents (HA2 peptides) (Xu Y. et al., 2018). Furthermore, to achieve targeted transport and drug release from NPs, many multifunctional NPs with improved properties such as active targeting, good aggregation, cellular uptake into lesion sites, pH sensitivity and thermal sensitivity have been developed (Blanco et al., 2015). Recent advances in material science have led to some new strategies balancing the degradation and drug release kinetics. Moreover, the size, shape and charge of NPs can be manipulated to improve the stability and efficiency of drug delivery systems.

## Applications of Oral Anti-CRC Nanotherapeutics

Several oral NPs are under development or in clinical trials. NPs are characterized by high solubility, high stability, controlled release, improved pharmacokinetics, and preferential accumulation in tumor tissues (Dadwal et al., 2018). The following section summarizes oral NPs developed for the treatment of CRC and classifies them into several types: lipid-based NPs, polymeric NPs, inorganic NPs, ligand-conjugated NPs, plant-derived NPs and stimuli-responsive NPs.

### Lipid-Based Nanoparticles

**FIGURE 3:**
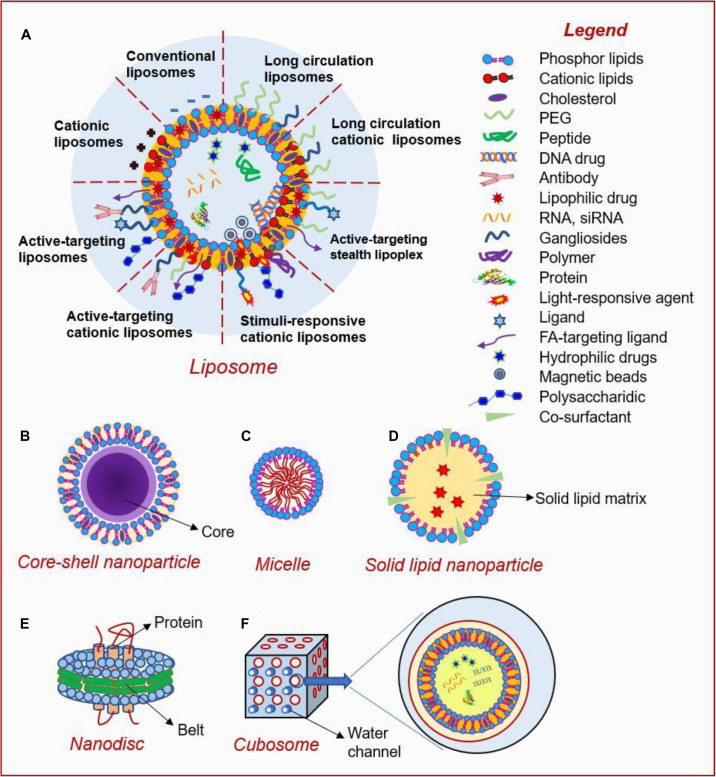
Types and structures of lipid-based nanoparticles (Yang and Merlin, 2020). Current lipid-based nanoplatforms include: **(A)** liposome, **(B)** core-shell nanoparticle, **(C)** micelle, **(D)** solid lipid nanoparticle, **(E)** nanodisc, and **(F)** cubosome.

Lipid-based nanoparticles (LNPs), especially liposomes, are promising platforms for cancer therapy, due to their good biodegradability, biocompatibility, structural simplicity, and tailorable functionality (Tang W. L. et al., 2018). LNPs can be basically divided into the following seven categories: liposome, core-shell NP, micelle, solid lipid nanoparticle (SLN), nanodisc and cubosome. The surface of liposomes can be modified by incorporating various functional motifs. LNPs are widely used not only because of their varieties, but also because they can be loaded with a variety of hydrophobic and hydrophilic therapeutic agents including chemotherapeutics, peptides, proteins, DNA, and RNA as shown in Figure 3 (Yang and Merlin, 2020). In this section, we focused on the current advances and applications of solid lipid NPs and liposomes.

Solid lipid NPs comprise lipids with low melting point, and varieties of surfactants and/or co-surfactants (Tapeinos et al., 2017). Selection and ratio of lipid and surfactant types determine the size, potential, stability, and drug loading and release profile of lipid NPs.

Solid lipid NPs are used to improve delivery of drugs with poor water solubility. For example, quercetin, an antioxidant found in onions, shows potent anti-tumor activities against CRC; however, it has poor water solubility. Li et al. (2009) designed quercetin-loaded solid lipid nanoparticles (QT-SLNs) using emulsification and low-temperature solidification method. The QT-SLNs showed longer Tmax and mean residence time (MRT), and improved relative bioavailability, implying that SLNs are effective oral delivery carriers for these hydrophobic agents.

Similarly, Shen et al. (2019) developed doxorubicin and superparamagnetic iron oxide NPs-loaded SLN delivery system and decorated the surface of SLNs with folate (FA) and dextran. Modification of these SLNs improved targeting capacity to cancer cells and facilitated its cellular uptake. *In vivo* experiments showed that the SLNs effectively inhibited primary tumor and metastatic burden, with lower systemic toxicity.

Liposomes can be classified into three types based on size and lamellarity. These classes include: small unilamellar vesicles (SUVs), large unilamellar vesicles (LUVs), and multilamellar vesicles (MLVs) (Yingchoncharoen et al., 2016). Particle sizes of SUVs range from 25 to 50 nm, and LUVs are larger than 100 nm in size, whereas sizes of MLVs ranges between 0.05 and 10 μm and are composed of multilayer phospholipid bilayer. In general, hydrophobic drugs are located in the phospholipid bilayer of the liposomes, whereas hydrophilic drugs are non-covalently trapped in the liposome cavity.

Batist et al. (2009) designed a novel liposome-encapsulated formulation of irinotecan and floxuridine, named CPX-1, and optimized the drug ratios to achieve therapeutic synergy. The codelivery system not only effectively maintained the high drug concentrations in blood circulation after systemically administered, but also showed high anti-tumor activity in CRC patients. Currently, a phase II trial for evaluating efficacy and safety of CPX-1 for CRC treatment is ongoing.

The therapeutic outcomes of several liposome-based chemotherapies are currently under clinical trials. OSI-7904L, a liposomal formulation of a non-competitive thymidylate synthase inhibitor (TSI), was designed for use in patients with advanced solid tumors (Beutel et al., 2005; Clamp et al., 2008; Ricart et al., 2008). In addition, liposomal DACH platinum L-NDDP, a liposomal formulation of *cis*-bis-neodecanoato-*trans*-R, R-1,2-diaminocyclohexane platinum (II) showed excellent anti-tumor activity in patients (Dragovich et al., 2006).

In addition, some natural lipid molecules have been used for drug derivatization and lipid nanoparticle preparation. For example, covalent conjugation of squalene (a natural lipid precursor) with different biologically active molecules enabled the squalenoylated prodrugs to self-assemble in aqueous media. Using this technology, Kotelevets et al. (2017) developed squalene-based nanoparticles loaded with cisplatin (SQ-CDDP NP) for oral delivery, thus significantly improving cisplatin efficacy. Treatment of CRC with cisplatin is associated with severe adverse effects and high risk of drug-resistance (Saber et al., 2018). The use of SQ-CDDP NPs enhanced the drug activity relative to its free drug form and circumvented the drug resistance mechanisms, partly due to facilitated cellular uptake. Inspired by these works, our group developed “PUFAylation” technology (Wu et al., 2020). Briefly, this strategy linked chemical drugs to polyunsaturated fatty acids (PUFAs). Such prodrug could self-assemble in aqueous media without exogenous excipients, hence provided effective anti-tumor effects.

In conclusion, lipid-based NPs are among the most promising drug delivery platforms given their biocompatibility, biocompatibility and extended functionality. They improve drug solubility, delivery efficiency, safety and efficacy making them ideal for cancer treatment.

### Polymeric Nanoparticles

**FIGURE 4:**
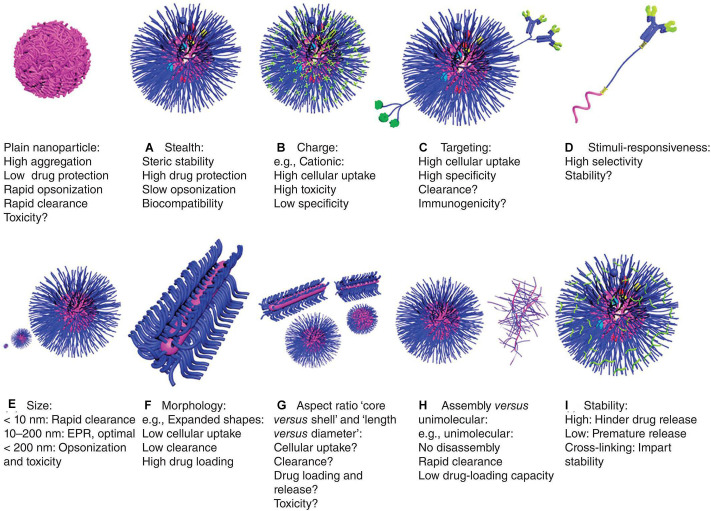
Characteristics of polymeric nanoparticles (Elsabahy and Wooley, 2012). The characteristics of polymer nanoparticles have great impact on their behavior *in vitro* and *in vivo*, including: **(A)** stealth: they have biocompatibility and stability, encapsulating drugs, which protect the nanoparticles from clearance by cells, but it may also reduce cell uptake and endosomal escape, **(B)** charge: cationic property can enhance cell uptake and endosomal escape, but their tissue distribution and toxicity are uncontrolled, **(C)** targeting: they have enhanced cellular uptake and specificity, but sometimes with accelerated clearance and/or immunogenicity, **(D)** stimuli-responsiveness: they can release drugs selectively at specific sites, but the stability and responsiveness of the materials may differ in physiological and pathological conditions, leading to premature drug release, **(E)** size: a size of about 100 nm is optimal for drug delivery, which is large enough to avoid being cleared by the kidneys, and small enough to reduce cell clearance and toxicity, **(F)** morphology: expanded morphology improves drug-loading capacity and reduces cellular uptake and clearance, **(G)** aspect ratio: the volume, length and diameter ratio of shell vs. core can greatly affect drug delivery efficiency and toxicity, **(H)** assembly vs. unimolecular structures: unimolecular structures are relatively more stable, but have poor drug-loading capacity and can be easily cleared, and **(I)** stability: stability of nanoparticles in circulation and physiological barriers and release at specific sites can be achieved by means of cross-linking.

Polymeric materials have been used in various pharmaceutical and biotechnology products for over 50 years. Polymer NPs for drug delivery have several advantages including: (1) high *in vitro* and *in vivo* stability, (2) ability to achieve targeted release in different parts of the body, (3) ability to load DNA, RNA and proteins and prevent their degradation, (4) ease of modification by ligands (Elsabahy and Wooley, 2012; Kamaly et al., 2012; Bao et al., 2013; Banik et al., 2016). Some physiochemical properties of therapeutic delivery systems such as, stealth, charge, targeting, stimuli-responsiveness, particle size, morphology, aspect ratio core vs. shell and length vs. diameter, assembly vs. unimolecular and stability can be tailored to make them more effective and reduce their toxic effects (Figure 4; Elsabahy and Wooley, 2012). For instance, in terms of charge, cationic NPs which exhibit high cellular uptake, high cytotoxicity, and low specificity have been developed. In this section, several classes of polymer materials are described.

Chitosan is a bioactive polymer, which is produced by alkaline deacetylation of chitin, a component of the exoskeleton of crustaceans (Prabaharan, 2015). It is widely used for drug conjugation or particle surface cloaking due to its high biocompatibility, biodegradability, cell membrane penetrability, low immunogenicity and low toxicity characteristics. Chitosan NPs are positively charged and can easily contact with cell membranes, which may facilitate the NP internalization into cancer cells (Narayanan et al., 2014; Zhang E. et al., 2019). Urbanska et al. (2012) encapsulated oxaliplatin in pH sensitive alginate microspheres with mucoadhesive chitosan coated. Oxaliplatin was loaded into NPs composed of lipid like polymeric molecules, and then these NPs were encapsulated in micro-sized alginate-based particles. The microparticles effectively passed through the stomach and targeted the intestinal tract, thus effectively alleviating tumor progression and morbidity. Hosseinzadeh et al. (2012) developed gemcitabine-loaded NPs through ionic gelation method using chitosan and Pluronic^®^ F-127 as a carrier. The gemcitabine-loaded NPs showed controlled release profile and had significantly higher cytotoxicity effect compared with free drugs. Apart from these chemotherapeutic agents, chitosan NPs can also be used to deliver natural molecules. For example, curcumin-loaded NPs were previously prepared in a similar manner as gemcitabine-loaded NPs (Chuah et al., 2013). Besides, Wen et al. (2018) developed core-sheath electrospun fiber mat containing quercetin-loaded chitosan NPs (Q-loaded EFM), which improved oral bioavailability and colonic targeting of quercetin, a bioactive flavonoid which can inhibit the progression of cancers (Rauf et al., 2018).

**FIGURE 5:**
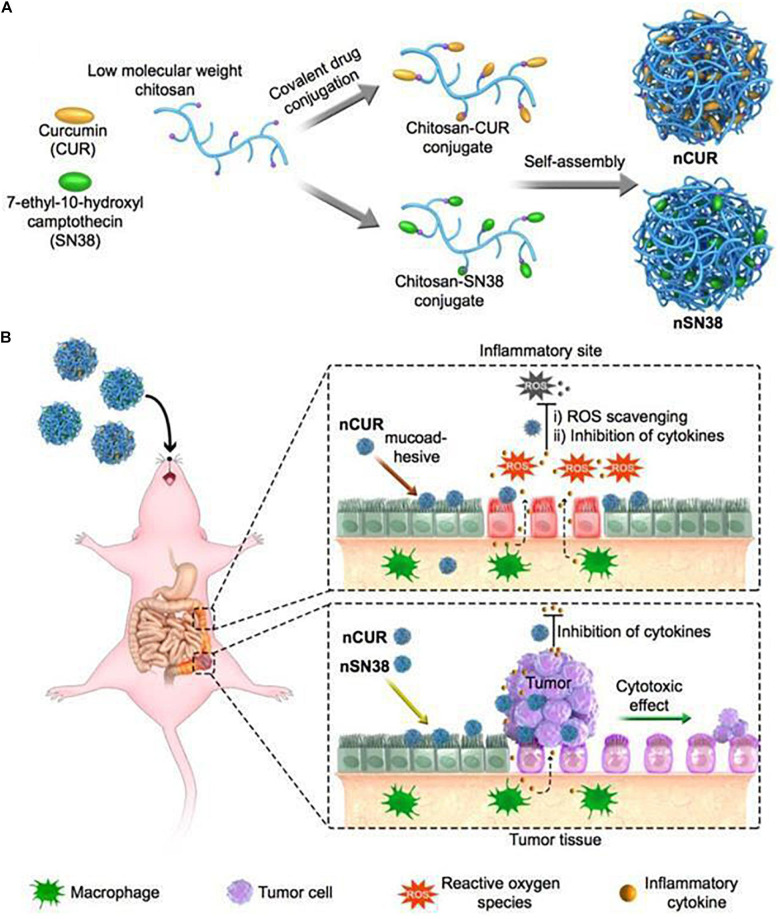
A scheme of self-assembly of chitosan-drug conjugates to form NPs and their oral administration for CAC treatment (Han et al., 2019). **(A)** Curcumin and SN38 were individually tethered to carboxylated chitosan by a self-hydrolyzable ester linkage. This formed amphiphile construct could be self-assembled in aqueous media and was suitable for oral administration. **(B)** Using a preclinical CAC mouse model, the anti-inflammatory nCUR and cytotoxic nSN38 nanoparticles could accumulate and release drugs in inflamed intestinal tissues and tumor tissues after oral administration, acting as an inhibitor of inflammation and tumor growth.

The development of CRC is often associated with chronic inflammation. Therefore, chitosan NPs can also be used to simultaneously co-deliver anti-inflammatory drugs and chemotherapies. Han et al. (2019) chemically engineered curcumin (CUR) and 7-ethyl-10-hydroxycamptothecin (SN38) into hydrophilic mucoadhesive chitosan to generate chitosan-drug amphiphiles for treatment of inflammatory bowel diseases (IBDs) and colitis-associated colorectal cancer (CAC) as shown in Figure 5. In this study, CUR and SN38 were attached to carboxylated chitosan by the hydrolyzable ester bond. The attachment of hydrophobic agents rendered the conjugates with the ability to self-assemble into stable and bioadhesive NPs for oral administration. Upon oral gavage, these NPs effectively accumulated in inflamed tissues and tumor sites, and adhered closely to intestinal villi. Subsequently, the NPs released CUR and SN38, exerting anti-inflammatory and anti-tumor effects, respectively.

Poly (_*D,L*_-lactic-co-glycolic acid) (PLGA)-based nanocarriers have been used for drug delivery. PLGA-based NPs are characterized by good biocompatibility and controlled biodegradation for effective delivery of drugs and macromolecules (Dechy-Cabaret et al., 2004). Yang et al. (2020) prepared PLGA/PLA-PEG-FA NPs (NP-PEG-FA/17-AAG) which improved the oral bioavailability of 17-AAG, an inhibitor of HSP that effectively treats ulcerative colitis and CAC. Results from the study indicated that NP-PEG-FA/17-AAG was effectively internalized by the inflamed bowel tissue of the mice, resulting in higher therapeutic efficacy, even at low doses.

Poly(ethylene glycol)-*block*-poly(_*D,L*_-lactic acid) (PEG-PLA) is a di-block copolymer. Many drugs can be tethered to the polymers and form polymer-drug conjugates. The amphiphilicity endowed by this approach makes them self-assembling in aqueous medium. TNP-470, an analog of fumagillin, is one of the first antiangiogenic drugs to undergo clinical trials. Clinical use of TNP-470 is limited due to poor oral availability and extremely short plasma half-life. Benny et al. (2008) developed an oral formulation of TNP-470, named Lodamin. TNP-470 was conjugated to PEG-PLA, forming polymeric nanomicelles, which had anti-tumor and anti-metastasis effects. Cell experiments showed that Lodamin was taken up by endothelial cells through endocytosis and retained its original anti-angiogenesis activity.

Several other kinds of materials have also been developed for preparation of orally deliverable NPs. For example, eudragit S100 is a pH-responsive enteric material used for site-specific delivery. 5-fluorouracil (5-FU) is one of the first-line drugs used for the treatment of CRC. 5-FU was originally administered intravenously; however, it is degraded rapidly in systemic circulation. In some studies, 5-FU was modified in different platforms for oral delivery to improve its anti-tumor effect and reduce toxicity (Miura et al., 2017). In a study designed by Subudhi et al. (2015), 5-FU was encapsulated into Eudragit S100 coated Citrus Pectin Nanoparticles (E-CPNs) to improve oral delivery of 5-FU. The E-CPNs released 5-FU in a pH-dependent manner and showed high safety, specificity and effectiveness *in vivo*. Moreover, citrus pectin, a ligand of galectin-3 receptors that is overexpressed on CRC cells, enhances the ability to target cancer cells. Additionally, irinotecan-loaded microbeads were designed using folic acid-grafted solid lipid nanoparticles and coated with Eudragit S100 to achieve pH-responsive drug release (Rajpoot and Jain, 2020).

Poly(acrylic acid) (PAA) is another pH-responsive polymer commonly used to improve water solubility of materials. Tian et al. (2017) capped mesoporous silica SBA-15 with PAA through a facile graft-onto approach. In this delivery system, SBA-15 acted as a gatekeeper which controlled drug transport in and out of the pore channels. By loading DOX into PAA/SBA-15, water solubility of DOX was significantly increased by loading DOX into PAA/SBA-15. In the stomach (pH = 2.0), DOX molecules were encapsulated in the pore channels because the pore outlets were capped with collapsed PAA. However, in the colon (pH = 7.6), it showed rapid release due to removal of the capping.

### Inorganic Nanoparticles

**FIGURE 6:**
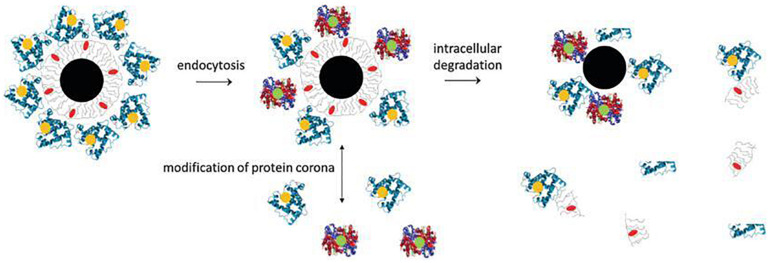
Composition and *in vivo* degradation of a tupical inorganic NP (Feliu et al., 2016). A typical inorganic NP comprises of an inorganic core (black), a engineered surface (gray) and a shell of adsorbed biological molecules (blue). During the degradation process, the nanoparticles may break down into individual components. The inorganic core may begin to decompose first, changing its physical and morphological characteristics. The engineered organic coating can also be partly removed by intracellular degradation.

Inorganic NPs, such as Metal NPs and Silica NPs, can be used as drugs, imaging agents, gene carriers, sensors and antiseptics (Sekhon and Kamboj, 2010). Advances in inorganic NPs in biomedicines provides a promising way to develop novel imaging and drug delivery systems as these NPs can be used for a variety of applications (Tee et al., 2019). Based on materials used and shapes, inorganic nanoparticles can be classified into: spherical gold, gold nanorod, gold nanocage, gold nanoshell, silver NPs, hafnium oxide, gadolinium oxide, mesoporous silica NPs, quantum dots, iron oxide, carbon nanotubes and carbon dots. A typical inorganic NP can be divided into three parts: an inorganic core, the engineered surface coating and a shell of adsorbed biological molecules. The fate of typical inorganic NPs after being administrated is shown in Figure 6 (Feliu et al., 2016). In short, plain inorganic cores, if not coated with an organic layer, will agglomerate under physiological environments. NPs coated with organic layers exhibit better biocompatibility and have good cell adhesion and cellular uptake properties. Once internalized, the physical and morphological characteristics of NPs are altered and their inorganic core is degraded. The organic layer can also be cleared by intracellular degradation or protein corona modification. In this section, we described the design and modification of metal NPs and silica NPs in detail.

Metal based NPs are widely used for treatment of CRC, as they are characterized by high stability and possibility of large-scale production avoiding organic solvents (Klebowski et al., 2018). Silver-based nanoparticles (AgNPs) are widely used in various fields, including medicine, food, health management, consumption and industry, due to their unique optical, electrical, and thermal and biological properties (Li W. R. et al., 2011; Zhang X. F. et al., 2016). Ranjan et al. (2013) synthesized starch-capped silver-based NPs. These NPs promoted apoptosis of HCT116 cells in a p53-dependent manner, by inducing increase in p53, p21 and Caspases 3, 8, and 9, and decrease in AKT and NF-κB (Satapathy et al., 2013). These metal-based NPs can be modified to improve effectiveness in drug delivery. Ghalandari et al. (2014) developed β-lactoglobulin (β-LG) NPs for oral delivery of oxali-palladium as a metal-based drug against colon cancer. This study reports that β-LG NPs containing oxali-palladium complexed with low methoxyl pectin (LMP) is a promising candidate for improving oral drug delivery for colon cancer treatment.

Furthermore, silica is commonly used in making inorganic NPs. Mesoporous silica nanoparticles (MSNs) were first reported in early 1900s (Kresge et al., 1992), and they are promising candidates for drug delivery in biomedical field. They effectively treats cancer cells due to the following properties: (1) they have large surface area and pore volume thus drugs can be adsorbed and loaded effectively, (2) they have mesoporous structure and adjustable pore size ensuring controlled release of drugs, (3) they have a surface that can be easily modified to achieve targeted therapy, (4) they are associated with good biosafety, biodegradability, distribution and excretion evaluations characteristics, (5) they allow combination with magnetic and/or luminescent materials, therefore bioimaging can be performed during drug administration (Wang Y. et al., 2015).

Liu W. et al. (2018) developed 5-FU@MSN-NH2/GC, galactosylated chitosans (GCs) based on mesoporous silica nanoparticle (MSN-NH2) which was loaded with 5-fluorouracil (5-FU) and functionalized with amino. These NPs showed high specificity in recognizing and binding to galactin receptors on cancer cells, thus promoting apoptosis of SW620 cells (Liu W. et al., 2018). In addition, these MSNs can be further modified. Kumar et al. (2017) developed a MSN-based enzyme-responsive materials which showed specific targeting of colon cancer cells. Guar gum, a natural carbohydrate polymer, was also used to cap drugs (GG-MSN). The NPs showed good anti-tumor effect in colon cancer cell lines. Moreover, Nguyen et al. (2017) loaded prednisone into 3-aminopropyl-functionalized MSN (MCM-NH2) and then wrapped it with succinylated ε-polylysine (SPL). SPL is a polymer obtained by modification of ε-polylysine (EPL). SPL was used for the first time to control drug release at pH 5.5–7.4 (the pH of the colon), with high stability of NPs in the stomach and small intestine. In addition, SPL may interact with intestinal and cancer cells through electrostatic interactions thus increasing drug residence time at the target site.

### Ligand-Conjugated Nanoparticles

**FIGURE 7:**
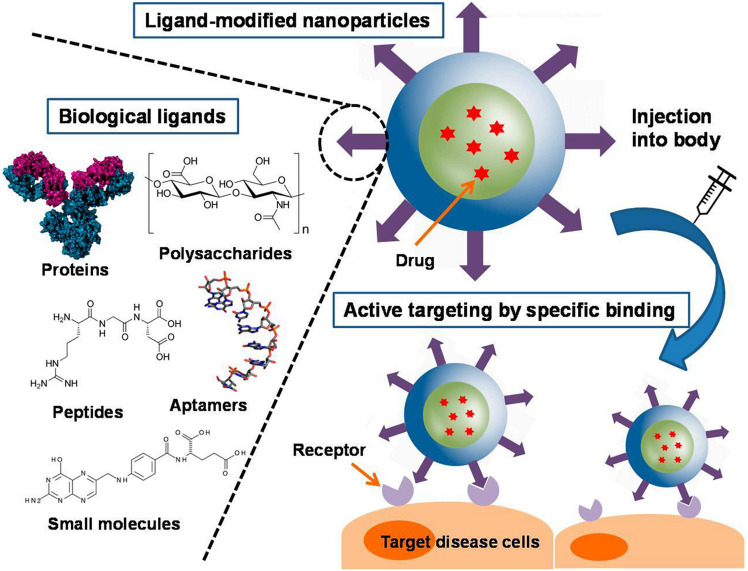
Different types of ligand-conjugated nanoparticles with various active molecules and their targeting mechanisms (Yoo et al., 2019). An increase in ligand density is advantageous for enhanced binding and cellular uptake. A variety of ligands, including proteins, polysaccharides, peptides, aptamers, and small molecules, are employed in functionalized nanoparticles. They can be physically adsorbed or chemically conjugated to the nanoparticles, or they can be tethered to the components of the nanoparticles before formation.

Targeting NPs to the lesion sites of CRC is important for their successful use. Many targeting ligands including small molecules, peptides, polysaccharides, antibodies, receptors, DNA, RNA have been identified and explored to facilitate active targeting of NPs, as shown in Figure 7 (Azharuddin et al., 2019; Yoo et al., 2019; Graczyk et al., 2020). These ligands can bind to specific receptors on the surface of the target cells, thus increasing the uptake of the NPs by the cells and ultimately improve therapeutic efficacy (Muhamad et al., 2018). In this section, we classified nanoparticles according to the types of modified ligands.

Hyaluronan (HA) is a natural polysaccharide consisting of N-acetylglucosamine and a β-glucuronic acid, which is one of the components of the extracellular matrix (Beldman et al., 2017). Its receptor, CD44, is highly expressed in several cancer types, therefore it is a potential target for cancer-targeting NPs. Xu et al. (2019) developed HA-NPs-PTC209, a colon cancer-targeted system to deliver the BMI-1 inhibitor, PTC209. PTC209 reverses stemness of CRC, thus reducing recurrence and metastasis of CRC. These HA-modified NPs have high affinity to CRC cells with high expression of CD44/CD168, and show effective targeting of tumor site. In addition, HA-CPT/CUR-NPs is a HA-modified NP developed by loading camptothecin (CPT)/curcumin (CUR) with HA-functionalized polymeric NPs (Xiao et al., 2015). This NP-based combination chemotherapy is efficient for colon cancer targeting.

Folic acid (FA) is used to modify anti-cancer drugs to improve specificity for human cancers which overexpress folate receptors (FRs). Moreover, folate binding protein, a glycosylphosphatidylinositol (GPI) that anchors cell surface receptor for folate, is overexpressed in some kinds of human cancers (Yoo and Park, 2004; Tang Y. et al., 2018). Desai et al. (2016) linked mesoporous silica NPs (MSNs) with poly(ethylene glycol) (PEG), poly(ethylene imine) (PEI) and FA in different combinations. The resulting MSNs were loaded with γ-secretase inhibitors of the Notch pathway for treatment of colon cancer. These modified MSNs specifically targeted the intestine and were easily internalized by intestinal epithelial cells, maintaining structural and functional integrity in the gastrointestinal environment. Rajpoot, Kuldeep, and Jain developed oxaliplatin-loaded FA-coupled solid lipid nanoparticles (SLNs) (OPSLNFs) formulations (Rajpoot and Jain, 2018). These OPSLNFs showed high entrapment efficiency, sustained drug release and high anti-cancer activity on HT-29 cell line.

Wheat germ agglutinin (WGA) is a dimer composed of two subunits and has four carbohydrate binding sites. It is derived from cereals and is a low immunogenic lectin (Xie et al., 2007). WGA recognizes certain types of cancer cells, therefore it is used to deliver NPs (Mo and Lim, 2005; Gao et al., 2007; Shen et al., 2011; Kuo et al., 2019). Wang and Huang (2019) integrated 5-fluorouracil (5-FU) and (−)-epigallocatechin-3-gallate (EGCG) into NPs, and then conjugated WGA to the surface of the nanoparticle through glutaraldehyde cross-linking (Wang R. et al., 2019). This colon cancer-targeted nanoparticle was named WGA-EF-NP. Glutaraldehyde cross-linking and positive charge of WGA-EF-NP results in smaller particle size and better stability *in vitro* and *in vivo* compared with EF-NP. Akl et al. (2019) designed curcumin-loaded PLGA NPs (C-PLGA NPs), and coated them with chitosan (CS), WGA and EGF analog peptides (GE11). Chitin promoted interaction of nanoparticles with cancer cells through non-specific electrostatic interactions, whereas WGA and GE11 promoted active targeting and specific recognition of cancer cells.

Song et al. (2019) developed Gd3^+^ -doped mesoporous hydroxyapatite NPs with polyacrylic acid (PAA) and anchored it to CS. Gadolinium was used as an MRI contrast agent in these NPs, PAA was a switch in response to pH, and PAA and CS ensured specific delivery of drugs to colon cancer tissues to avoid early release of drugs. In addition, these NPs could be loaded with chemotherapy drugs 5-FU and gefeitinib for localized targeted treatment of colon cancer. Zu et al. (2019) developed CS-functionalized camptothecin (CPT)-loaded polymeric NPs, with better pro-apoptotic ability and anti-colon cancer efficiency compared with non-targeted NPs. These CS-CPT-NPs showed a desirable hydrodynamic particle size, excellent monodispersity, narrow particle size distribution, electro-neutral surface, and good stability in biological environment, which improved their anti-cancer activities.

Alginic acid is a natural linear anionic polysaccharide which is formed from α-l-guluronic acid and β-d-mannuronic acid (Rhim et al., 2006; Zare et al., 2015). Alginic acid is characterized by good biodegradability, biocompatibility, non-toxicity and mild gelation properties, therefore, it is widely used in biomedical applications, such as surface modification of biomedical implants and drug delivery systems (Lee et al., 2009; Kolambkar et al., 2011; Srivastava et al., 2012). Victor et al. (2016) developed a nanoplatform with near-infrared fluorescence capability based on neodymium doped hydroxyapatite (HAN). The surface of these NPs was modified using alginic acid, resulting in NPs with appropriate particle size, needle shape, negative zeta potential, pH response, oral colonic targeting and were conducive to cell internalization. These NPs can be used to achieve early diagnosis of cancers, targeted cancer therapy and surveillance of colon cancer after oral administration.

**FIGURE 8:**
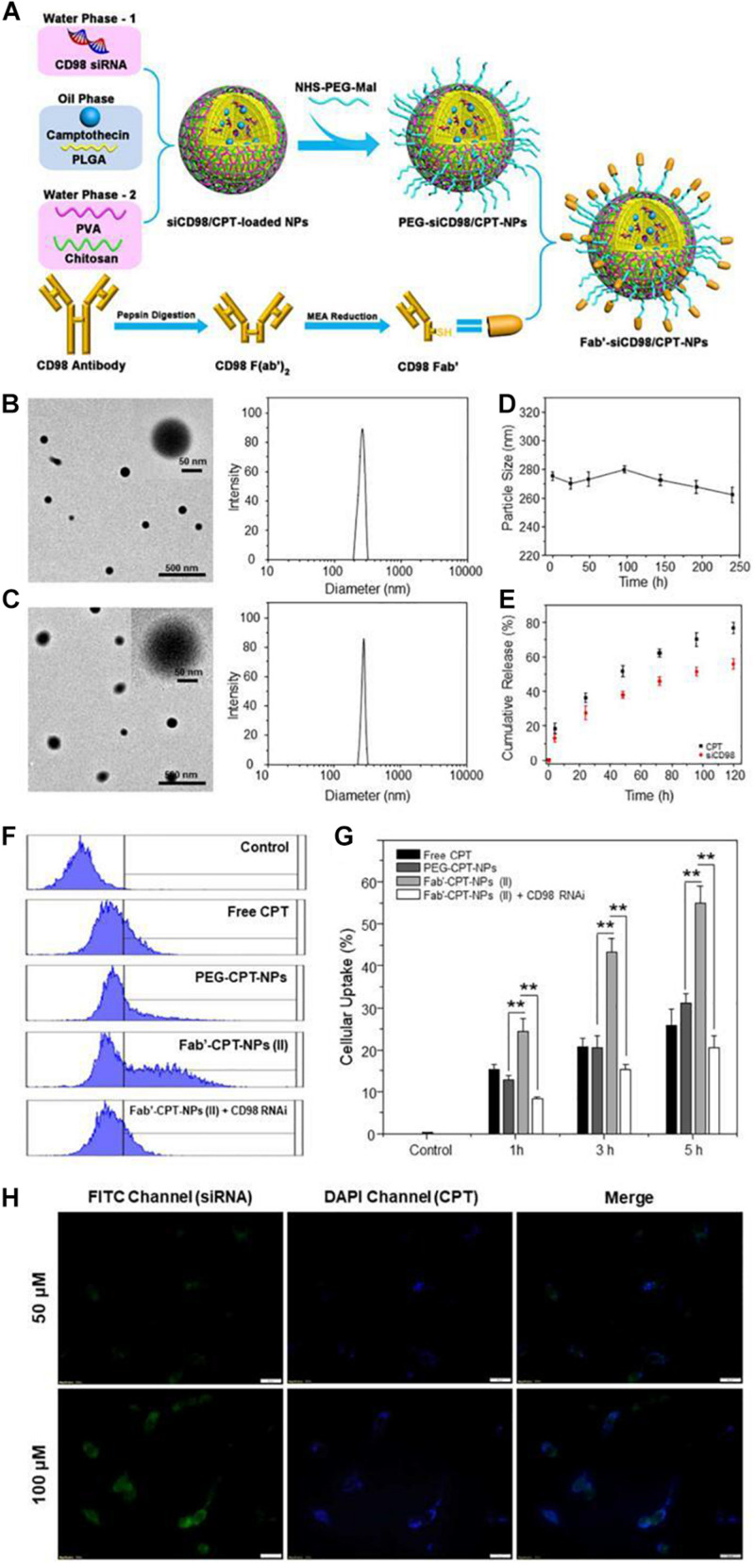
Fabrication, physicochemical characterization and *in vitro* targeting capacity of Fab’-functionalized nanoparticles (Elsabahy and Wooley, 2012). **(A)** A schematic illustration of preparation of Fab’-siCD98/CPT-NPs. Initially, Fab’-SH was generated from CD98 antibodies. Then MAL-siCD98/CPT-NPs were yielded by coating siCD98/CPT-NPs with MAL-PEG-NHS. Finally, MAL-siCD98/CPT-NPs were reacted with Fab’-SH to obtain II. **(B,C)** Representative transmission electron microscope images and size distribution of I and II, respectively. **(D)** The average particle size of II in deionized water after 240 h of storage at room temperature. **(E)** *In vitro* drug release profiles of siCD98 and CPT from II at 37°C. **(F)** Representative FCM histograms of fluorescence intensity of cells that were treated with I and II with same CPT concentration (32 μM) for 5 h. **(G)** Proportion of CPT-containing cells after being treated 1, 3, or 5 h as **(F)**. **(H)** Fluorescence microscopy images of Fab’-FITC-siRNA/CPT-NPs cellular uptake profiles in Colon-26 cells after 5 h treatment, with a scale bar of 20 μm. *Note: (I)PEG-siCD98/CPT-NPs, (II)Fab’-siCD98/CPT-NPs.*

Proteins and peptides are characterized by good biodegradability, biocompatibility and low immunogenicity properties, therefore, they are widely used to modify the surface of NPs. Modified proteins and peptides promote targeting of cancer tissues through receptor-ligand interaction. Colon cancer patients highly expresses CD98, therefore, Xiao et al. (2018) developed CD98 Fab’-functionalized NPs for co-delivery of camptothecin (CPT) and CD98 siRNA, which were highly effective in drug delivery to cancer tissues and showed improved anti-cancer effects as shown in Figure 8. Briefly, they coated siCD98/CPT-NPs with MAL-PEG-NHS to create MAL-siCD98/CPT-NPs. The resultant complex was reacted with Fab’-SH generated from CD98 antibodies, leading to the formation of Fab’-siCD98/CPT-NPs. The particle size, morphology, stability and cellular uptake profiles of the nanoparticles were characterized. The results showed that both nanoparticles exhibited good particle size distribution, and the PEG-siCD98/CPT-NPs were slightly smaller than Fab’-siCD98/CPT-NPs. Both nanoparticles showed solid spherical morphology as determined by transmission electron microscopy and were relatively stable in deionized water and in simulated colonic fluid. In terms of cell internalization, the cellular uptake efficiency of Fab’-CPT-NPs (II) was significantly lower in CD98-knockdown Colon-26 cells, demonstrating that CD98 might play important functions in cells. They further prepared NP-embedded hydrogel, which precisely and systematically controlled specific release of NPs in the colonic lumen and their internalization by target cancer cells.

Nucleic acids can also be attached to the surface of NPs to act as ligands. Aptamers, also known as chemical antibodies, are short-stranded RNA or single-stranded DNA that bind to targets with high specificity and affinity (Alshaer et al., 2018). They are characterized by low toxicity, low immunogenicity and ease of production (Zhou and Wang, 2006). Xie et al. (2016) developed mesoporous silica NPs (MSNs) with doxorubicin (DOX) loaded and modified with aptamer, which targets epithelial cell adhesion molecule (EpCAM), and named the NP Ap-MSN-DOX. These NPs specifically bound to EpCAM over-expressing SW620 colon cancer cells, thus enhancing cellular uptake and increasing cytotoxicity to SW620 cells.

### Plant-Derived Nanoparticles

Plant-derived natural NPs are used to overcome off-target cytotoxicity and high cost of mass production of traditional NPs. Nanocarriers derived from edible plants, such as ginger, grapefruit, broccoli, and lemon, can be used for drug delivery (Wang et al., 2013; Zhang et al., 2016a, b; Yang et al., 2018; Zhang M. et al., 2019). These nanocarriers have exosomal-like traits and can be applied in exosome-based drug delivery systems. Exosomes are cell-derived and widely distributed, therefore, exosome-based NPs are relatively non-toxic, non-immunogenic, highly efficient, highly specific and can be used as a powerful nanocarrier to deliver drugs. Some exosome-based NPs can be delivered orally to treat CRC (Wang J. et al., 2016).

Zhang et al. (2016a) isolated nanoparticles from edible ginger, which has several anti-inflammatory, anti-oxidative, and anti-tumor active constituents. These isolated NPs were exosome-like, with high levels of lipids, some proteins, miRNAs, and several bioactive constituents. This natural delivery mechanism ensures low toxicity and mass production of NPs. In a previous study, doxorubicin (Dox) was loaded with a nanovector made from ginger-derived lipids (GDNVs) (Zhang et al., 2016b). The drug-loaded nanovectors were effectively taken up by colon cancer cell lines, leading to the inhibition of cell proliferation and enhancement of cell apoptosis. In addition, conjugation of GDNVs and folic acid may enhance targeting of drugs to colon-26 tumors, hence improve efficacy.

### Stimuli-Responsive Nanoparticles

Tumor microenvironment consists of extracellular matrix (ECM), tumor-related immune cells, neuroendocrine (NE) cells, adipose cells, blood and lymphatic vascular networks, cytokines, stroma, and other signaling molecules (Chen F. et al., 2015; Wang et al., 2017). Some bone marrow-derived progenitor cells are recruited to the TME and differentiate into endothelial cells, pericytes, fibroblasts and other stromal cells, thus promoting malignant progression of the tumor (Joyce and Pollard, 2009; Hanahan and Weinberg, 2011). In addition, TME plays an important factor in regulation of cancer cell metabolism (Cairns et al., 2011). Therefore, a better understanding of the interaction between TME, cancer cells and drugs is important in treatment of tumors.

**FIGURE 9:**
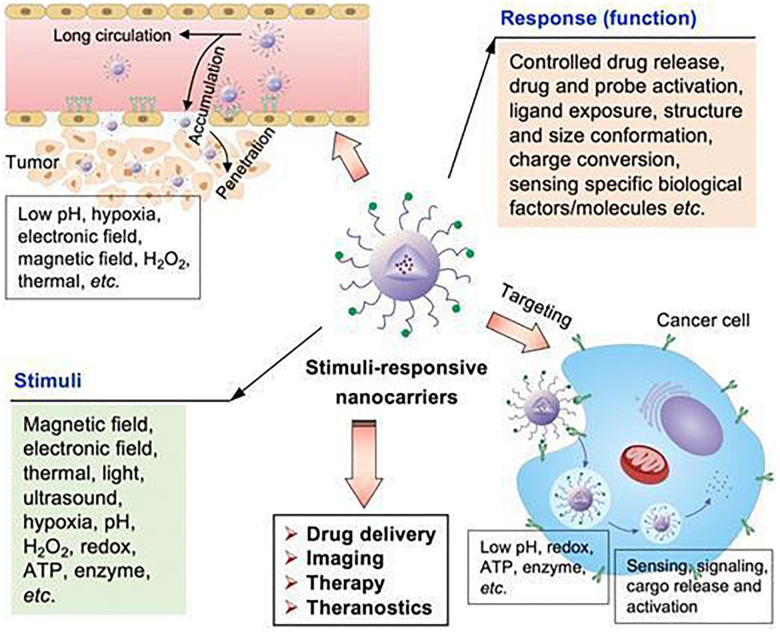
The stimuli-sensitive functions of stimuli-responsive nanocarriers (Cairns et al., 2011). Nanoparticles can accumulate in tumor sites, responding to internal and external stimuli. Internal stimuli include: pH, redox, ATP, enzyme, and hypoxia etc., while external stimuli include electronic field, magnetic field, thermal, light and ultrasound. The stimuli-responsive characteristic promotes the release of drugs in tumor sites to achieve accurate diagnosis and treatment of tumors.

Tumor microenvironment is characterized by acidity, hypoxia and thermal stability properties, which are conducive for development of stimuli-responsive nanoparticles. Therefore, these NPs ensure tumor targeted release as they are inactive in blood circulation and under normal physiological conditions (Yu et al., 2014; Du et al., 2015; An et al., 2016; Kang et al., 2017; Wu and Dai, 2017); once they arrive at the tumor site through passive or active targeting, they can be activated to release drugs in response to the characteristics of TME, thus achieving specific drug release and reducing adverse reactions. Interactions between stimuli-responsive nanocarriers and TME are shown in Figure 9 (Cairns et al., 2011). Stimuli-responsive nanocarriers can be used in drug delivery systems, imaging, therapy and theranostics. These carriers respond to internal stimuli (low pH, hypoxia, ATP, enzyme, redox, etc.) and external stimuli (magnetic field, electronic field, thermal, light, ultrasound, etc.). Given these properties, they can control drug release, drug and probe activation, ligand exposure, structure and size conformation, charge conversion, and response to specific biological molecules, thereby improving site-specific delivery.

Reactive oxygen species (ROS)-responsive NPs are common types of stimuli-responsive NPs. Vong et al. (2015) developed an oral nanotherapy using a redox nanoparticle RNP(O) by self-assembly of MeO-PEG-b-PMOT. In the core of RNP(O) is nitroxide radicals, acting as ROS scavengers. Oral administration of RNP(O) and combination with irinotecan significantly improved therapeutic efficacy for CAC in mice. Furthermore, orally administered RNP(O) was effectively internalized in cancer cells compared with normal cells, preventing undesired adverse effects and toxicity.

Several other redox nanoparticles have been developed for treatment of CRC. Vong et al. (2017) designed silica-containing redox nanoparticle (siRNP) with the ability to scavenge ROS. They also used BNS-22, a hydrophobic anticancer compound, as a novel oral nanocarrier for treatment of colitis-associated colorectal cancer.

OCD is also an oxidation-responsive material, obtained by hydrophobic linkage of β-cyclodextrin (β-CD) with an oxidation-labile group (Zhang et al., 2020). Zhang Q. et al. (2019) loaded a chemotherapeutic agent CPT-11 into OCD NP, called CPT-11/OCD NP. These NPs significantly reduced oxidative stress and inhibited inflammation in cancer cells. After oral administration, angiogenesis and tumor growth of colitis-associated colon cancer were significantly inhibited, especially in the inflammatory phase.

In addition, NPs also respond to pH and thermal changes in the intestinal environment to achieve specific drug release which improves CRC treatment. The research by Xiguang Chen designed DAHBCs NPs which were pH- and thermo-responsive. Hydroxybutyl chitosan (HBC) was prepared by conjugating hydroxybutyl groups to the backbone of chitosan, which served as a thermo-responsive polymer. HBC was then modified with deoxycholic acid (DOCA) to form an amphiphilic polymer named DAHBCs NPs with the ability to self-assemble in aqueous medium (Yang et al., 2013). Curcumin (CUR) was then loaded into DAHBCs NPs, resulting in optimal drug loading ratio (Wang F. et al., 2016). These NPs exhibited pH-responsive expansion and thermo-responsive shrinkage at low pH and 37°C in the stomach, thus remaining stable (Wang T. et al., 2019). In the intestinal environment, only a thermo-responsive shrinkage effect was observed, therefore, the drug was released in large quantities in the intestinal tract, thus exerting excellent anti-tumor effect.

## Summary and Perspectives

The incidence and mortality rates of CRC have been increasing annually. This necessitates the search for highly efficient and less toxic treatment options for CRC patients. Conventional treatment regimens have limited efficacy, low targeting efficiency, and numerous side effects. Nanotherapeutics has emerged as a suitable alternative for successful treatment of CRC, and various formulations of nanoparticles have been used to design drug delivery systems. Of all drug delivery strategies, oral administration is considered to be the most common and acceptable form, due to their advantages of reduced pain, ease of ingestion and high patient compliance. For drugs with poor stability, solubility and bioavailability, encapsulating them into nanoparticles might make them suitable for oral administration. In this review, we discussed the types, composition, and effects of oral nano-drugs used for CRC treatment.

Numerous studies have attempted to improve the medicinal properties of insoluble anti-tumor drugs. Some of the approaches used to achieve this include strategies to avoiding acid or enzymatic degradation of drugs in the gastric environment, increasing drug retention in the gastrointestinal tract and absorption in intestinal epithelial cells. Currently, several nano-drugs are available for clinical use. Some researchers have reviewed the nanodrugs approved for clinical use and clinical trials testing their efficacy in the treatment of CRC (Bobo et al., 2016; Caster et al., 2017; Ventola, 2017; Anselmo and Mitragotri, 2019). Table 2 summarizes the oral nanotherapeutics undergoing clinical trials for the treatment of CRC.

**TABLE 2:** Nanoparticles that are not clinically approved but are in clinical trials (including not yet recruiting, recruiting, or active) for treatment of CRC by oral administration.

ClinicalTrials.gov identifier (NCT number)	Intervention/treatment	Indication	Clinical trial status
NCT03774680	Drug: Cetuximab nanoparticles Drug: Oral approved anticancer drug	Colon cancer Colo-rectal Cancer	Phase 1
NCT02762981	Drug: CORT125134 with nab-paclitaxel Other names: Abraxane Nanoparticle albumin-bound paclitaxel Relacorilant	Triple-negative breast cancer Ovarian epithelial cancer Pancreatic cancer solid tumors	Recruitment status: Completed First posted: May 5, 2016 Last update posted: November 19, 2020
NCT00313599	Drug: lapatinib Drug: paclitaxel	Advanced solid tumor malignancies	Phase 1

However, compelling evidence indicates that the available nano-drug delivery systems are not sufficiently effective. Apart from biosafety and pharmacokinetics, the clinical application of nanoparticles can also be limited by various issues, including intellectual property, mass manufacturing, and overall cost-effectiveness compared with current therapies. Therefore, it is necessary to develop strategies to improve the critical characteristics of NPs so that they can be used in mainstream clinical practice to treat CRC. For example, numerous studies have shown that the adhesion of NPs to the mucus barrier limits the performance of nano-drugs. Hence, NPs that effectively penetrate the loosely adherent mucus layer and reach the firmly adherent layer have longer retention time which improves efficacy of the drugs. To simulate the absorption and transport of NPs in the intestinal tract, some cell and tissue models have been established. For example, Caco-2 cell, a human colon cancer cell line, is commonly used as a model of human intestinal epithelium without a mucus layer, with a relatively lower endocytotic activity. The Ussing chamber, an advanced *in vitro* technique used to study the transepithelial transport of the gut, allows us to investigate permeability and other physiological properties of the intestinal epithelium. The Ussing chamber can also be used as a model to study intestinal epithelial absorption of small molecule drugs *in vivo*. To accelerate the clinical application of nanoparticles, future researches should develop strategies to reduce the complexity of nanoparticle synthesis and select the optimal dosage form and formulations that are most suitable for clinical use.

## Author Contributions

KY, HW, and BX wrote and revised the manuscript. BB, XG, and YX helped collect materials. HW and BX supervised and finalized this manuscript.

## Conflict of Interest

The authors declare that the research was conducted in the absence of any commercial or financial relationships that could be construed as a potential conflict of interest.
